# Analysis and prediction of antibacterial peptides

**DOI:** 10.1186/1471-2105-8-263

**Published:** 2007-07-23

**Authors:** Sneh Lata, BK Sharma, GPS Raghava

**Affiliations:** 1 Institute of Microbial Technology, Sector39A, Chandigarh, India

## Abstract

**Background:**

Antibacterial peptides are important components of the innate immune system, used by the host to protect itself from different types of pathogenic bacteria. Over the last few decades, the search for new drugs and drug targets has prompted an interest in these antibacterial peptides. We analyzed 486 antibacterial peptides, obtained from antimicrobial peptide database APD, in order to understand the preference of amino acid residues at specific positions in these peptides.

**Results:**

It was observed that certain types of residues are preferred over others in antibacterial peptides, particularly at the N and C terminus. These observations encouraged us to develop a method for predicting antibacterial peptides in proteins from their amino acid sequence. First, the N-terminal residues were used for predicting antibacterial peptides using Artificial Neural Network (ANN), Quantitative Matrices (QM) and Support Vector Machine (SVM), which resulted in an accuracy of 83.63%, 84.78% and 87.85%, respectively. Then, the C-terminal residues were used for developing prediction methods, which resulted in an accuracy of 77.34%, 82.03% and 85.16% using ANN, QM and SVM, respectively. Finally, ANN, QM and SVM models were developed using N and C terminal residues, which achieved an accuracy of 88.17%, 90.37% and 92.11%, respectively. All the models developed in this study were evaluated using five-fold cross validation technique. These models were also tested on an independent or blind dataset.

**Conclusion:**

Among antibacterial peptides, there is preference for certain residues at N and C termini, which helps to demarcate them from non-antibacterial peptides. Both the termini play a crucial role in imparting the antibacterial property to these peptides. Among the methods developed, SVM shows the best performance in predicting antibacterial peptides followed by QM and ANN, in that order. AntiBP (Antibacterial peptides) will help in discovering efficacious antibacterial peptides, which we hope will prove to be a boon to combat the dreadful antibiotic resistant bacteria. A user friendly web server has also been developed to help the biological community, which is accessible at .

## Background

In the past few decades, a large number of bacterial strains have evolved ways to adapt or become resistant to the currently available antibiotics [1]. Researchers are focusing on alternative drugs based on antimicrobial peptides, which play an important role in innate immunity. As part of innate immune system, antimicrobial peptides provide protection against a wide variety of microorganisms in both vertebrates and invertebrates [2-10]. These peptides are very diverse with respect to amino acid sequence and secondary structure but share certain properties, such as affinity for the negatively charged phospholipids that are present on the outer surfaces of the cytoplasmic membrane of many microbial species. These peptides are ubiquitous, simple and effective factors acting within the innate immune system. Their short length and fast & efficient action against microbes has made them potential candidates as peptide drugs [4,11]. Several peptides and their derivatives have already passed clinical trials successfully [12,13] and several others are considered as potential therapeutics [12].

Antimicrobial peptides have a broad spectrum of activity, including activity against bacteria, fungi, viruses, and even cancer cells [14]. Other than having pathogen-lytic activities, these peptides have other properties like anti-tumour activity; mitogen activity, or act as signalling molecules [14]. In addition, they have a number of biotechnological applications, e.g. in transgenic plants [15,16], in aquaculture, and as aerosol spray for patients of cystic fibrosis [17]. In the past extensive work has been done in the field of antibacterial peptides, describing their identification, characterization, mechanism of action etc. The information about these peptides has been collected and compiled; following are major databases on antimicrobial peptides I) ANTIMIC have around 1700 sequences [18] ii) AMSDb consists 804 antimicrobial peptides of eukaryotic origin [19], iii) Peptaibol consists of around 300 antibiotic peptides originated from fungal organisms [20] and iv) APD consists of detailed information for 525 antimicrobial peptides [21].

Recently, a HMM (Hidden Markov Model) based method has been developed for searching conserved motifs of β-defensin family in genome databases [22]. The antibacterial peptides have little sequence homology, despite common properties [12]. Thus it is difficult to develop method for predicting the antibacterial peptides based on similarity. Moreover, experimental methods for identification and designing of antibacterial peptides are costly, time consuming and resource intensive. Thus there is a need to develop computational tools for predicting antibacterial peptides, which could be used to design potent peptides against bacterial pathogens.

In the present study, a systematic attempt has been made to understand this important class of peptides and to develop an algorithm for predicting antibacterial peptides with high accuracy. First, we collected and analyzed antibacterial peptides in order to understand preference of residue type at different positions. We also compared composition of antibacterial and non-antibacterial peptides. We analyzed residues of both N and C termini as both play important role in their activity; for example, C-terminus is responsible for the membrane interaction and pore formation, while the N-terminal region is important in bacteria-specific interaction process [23]. Based on our observation, we developed our methods using both N-terminus and C-terminus approach. In this study, three popular classification techniques, namely, Quantitative Matrix (QM), Artificial Neural Network (ANN) and Support Vector Machine (SVM), have been used to predict antibacterial peptides.

## Results

### Analysis of the antibacterial peptides

**Figure 1:**
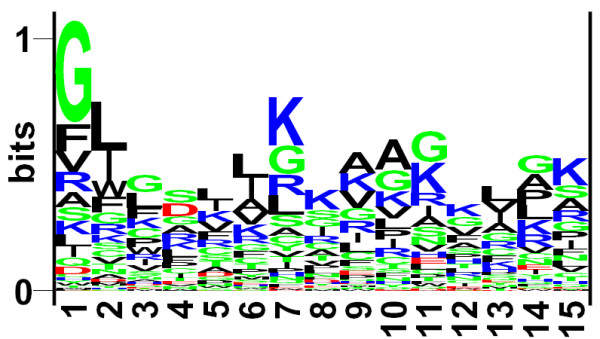
The figure depicts the sequence logo of first fifteen residues (N-terminus) of antibacterial peptides, where size of residue is proportional to its propensity

**Figure 2:**
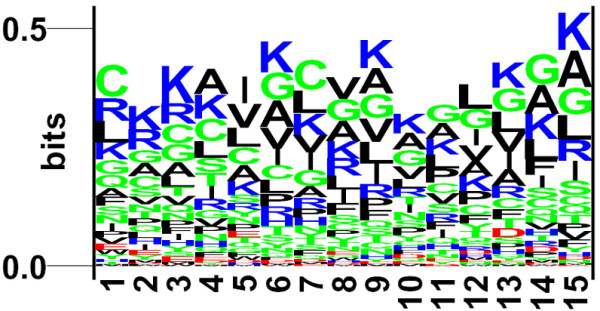
The figure depicts the sequence logo of last fifteen residues (C-terminus) of antibacterial peptides, where size of residue is proportional to its propensity.

The frequency of occurrence of all 20 natural amino acids at both termini was examined. It was observed that particular types of residues are preferred over others in anti-bacterial peptides. In order to demonstrate residue preference at different position of antibacterial peptides, sequence logos using plogo program [24] were generated. The sequence logos of 15 N-terminal and C-terminal residues are shown in Figure 1 and 2, respectively. As shown in Figure 1, certain residues are more abundant at specific positions, e.g., G, F, V, R at first position; L, I, W, F at 2^nd ^position etc. Overall antibacterial peptides are dominated by certain type of residues like G, R, K, L etc., being present at most of the positions. Similarly, certain residues are preferred at the C-terminus, for example residues K, G, C, and R are preferred at most of the positions.

In order to understand biasness of residues in antibacterial peptides, we computed frequency of each type of the residues in antibacterial and non-antibacterial peptides. As shown in Figure Additional file: 1 (Supplementary Material), residues G and F are significantly higher at first position of N-terminus of antibacterial peptides than that of non-antibacterial peptides. Similarly residues W, I, L, F are more frequent at the 2^nd ^position of N-terminus of antibacterial peptides in comparison to non-antibacterial peptides. We also computed polar, non-polar, positive charge residues etc. at different positions of antibacterial peptides and compared with non-bacterial peptides (See Figure S11 to S20). Though both termini possess higher frequency of positively charged residues (K and R), the difference between the frequency of these residues between the antibacterial and non-antibacterial peptides is higher at the C-terminus. The difference in the frequencies of the positively charged residues between the positive and negative examples increases from position 1 to 5 at the N-terminus, whereas it tends to decrease from position 1 to 5 at the C-terminus. While higher frequency of the positively charged residues may be helping the C-terminus to interact with the negatively charged bacterial membrane, positively charged residues at the N-terminus may help in the interaction between these peptides and intracellular components like DNA and RNA, thus hampering the crucial bacterial functions. The proportion of Cysteine is higher at the N-terminus also. The proportion of negatively charged amino acids (D and E) were also found to be very low, as expected, since these may interfere during the course of penetration of the negatively charged cell membrane of the bacteria.

#### Overall compositional biasness

**Figure 3:**
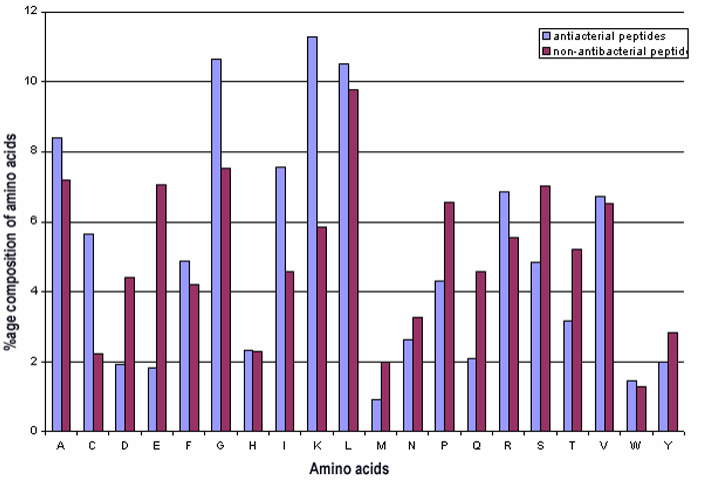
Overall amino acid composition of antibacterial vs non-antibacterial peptides. This figure represents a comparison of composition bias of various amino acids in antibacterial and non-antibacterial peptides.

As shown in the above analysis (Figure S1-S20), antibacterial peptides have preference for certain types of residues at certain positions. The next question is if the overall composition of these antibacterial is also different from that of non-antibacterial peptides. Thus we compared the overall amino acid composition of antibacterial and non-antibacterial peptides. As shown in Figure 3, certain residues like C, G, I and K are significantly higher in antibacterial peptides, as expected, since these residues are preferred at certain positions in antibacterial peptides. It is also noteworthy that residue R was not significantly higher in antibacterial peptides, though it was preferred at certain positions. Certain residues like D, E, S and P are not preferred in antibacterial peptides.

### Prediction of Antibacterial Peptides

#### Whole peptide

The prediction of antibacterial peptides is difficult, but very important. In the past attempts have been made, but these methods are subjective, based on physico-chemical property plots, than quantitative. The prediction of antibacterial peptides is difficult due to variation in the size of antibacterial peptides, since the machine learning techniques need fixed length pattern. Thus the question was how to circumvent this problem and develop a machine learning based method for predicting antibacterial peptides. As shown in Figure 3, amino acid composition of antibacterial peptides is significantly different from that of non-antibacterial peptides. In the past, methods have been developed based on machine learning techniques, using protein composition to classify the proteins (in spite of their variation in size), particularly for subcellular localization of proteins. Thus, we have developed a SVM based model using amino acid composition on our main dataset and achieved an accuracy of 89.04% with sensitivity of 89.92% and specificity of 88.09% (Table 1). Though we could predict antibacterial peptides with high accuracy using amino acid composition, we realised the weakness of this composition-based approach, since it is difficult to decide what length we should search in proteins as length of antibacterial peptides varied from 5 to ~80. Despite its better performance, it is impossible to use this approach for searching antibacterial peptides in genomes or proteins.

**Table 1:** The performance of SVM models using amino acid compositions of whole peptides and five residues of N-terminus. Last column shows performance of binary-based SVM model using first five residues of peptides.

	**Whole Peptide Composition**			**5 N-terminal residue Composition**			**5 N-terminal binary Pattern**		
	
**Threshold**	**Sen.**	**Spec.**	**Acc.**	**Sen.**	**Spec.**	**Acc.**	**Sen.**	**Spec.**	**Acc.**
-1	98.15	60.45	80.13	97.31	25.45	61.38	95.81	24.25	60.03
-0.9	97.94	63.60	81.53	95.21	30.54	62.87	94.31	30.54	62.43
-0.8	97.53	67.19	83.03	93.71	35.33	64.52	93.71	37.13	65.42
-0.7	97.12	70.56	84.43	91.62	39.82	65.72	91.62	43.11	67.37
-0.6	96.91	72.81	85.39	90.42	46.11	68.26	90.12	48.50	69.31
-0.5	96.30	76.18	86.68	89.22	50.60	69.91	86.83	54.19	70.51
-0.4	95.88	77.53	87.11	87.72	55.09	71.41	85.03	59.88	72.46
-0.3	94.65	80.90	88.08	84.73	58.98	71.86	82.34	64.97	73.65
-0.2	93.42	84.27	89.04	81.44	63.47	72.46	80.24	68.86	74.55
0	**89.92**	**88.09**	**89.04**	**73.65**	**72.75**	**73.20**	**74.25**	**74.85**	**74.55**
0.1	88.48	89.44	88.94	70.36	76.65	73.50	70.66	77.54	74.10
0.2	86.63	90.56	88.51	66.77	79.64	73.20	67.96	83.83	75.90
0.3	84.98	91.01	87.86	63.47	83.83	73.65	64.97	88.32	76.65
0.4	83.74	93.03	88.18	57.49	86.83	72.16	60.78	88.92	74.85
0.5	80.04	93.26	86.36	53.59	89.52	71.56	57.78	90.12	73.95
0.6	77.37	94.83	85.71	48.50	91.62	70.06	54.49	91.02	72.75
0.7	75.51	95.28	84.96	44.61	94.01	69.31	51.50	93.41	72.46
0.8	72.02	95.28	83.14	39.22	94.91	67.07	44.91	95.51	70.21
0.9	67.90	96.40	81.53	34.43	96.11	65.27	39.52	96.11	67.81
1	64.61	97.53	80.34	29.04	97.90	63.47	32.34	97.01	64.67

#### N-terminal approach

It has been observed that certain types of residues are preferred at the N-terminal regions of antibacterial peptides. Thus we made an attempt to develop a method for predicting antibacterial peptides using their N-terminal residues. We created a dataset NT5 which consists of 5 N-terminal residues of antibacterial and non-antibacterial peptides (See Material and Methods). Then we developed a SVM model using the amino acid composition of these N-terminal residues and achieved an accuracy of 73.20% with 73.65% sensitivity and 72.75% specificity (Table 1). The performance of this SVM module is much lower than of SVM module using composition of whole peptide, but this module can be used to scan antibacterial peptides in protein sequences. As we are using only first five residues here, it's possible to use these residues as binary pattern instead of composition. In binary pattern a vector of dimension 20 represents a residue, and for 5 residues the input vector of dimension is 100 (20 × 5). As shown in Table 1, we achieved an accuracy of 74.55% using the binary pattern based SVM model. Overall, the binary-based SVM model performs better than composition based model using first five N-terminal residues. Thus we extended our binary-based SVM models for predicting antibacterial peptides using more number of residues from the N-terminus. As shown in Table 2, we achieved an accuracy of 86.45% with specificity of 87.68% using binary-based SVM using the first 10 N-terminal residues. We got the best accuracy using first 15 N-terminal residues, which is 87.85% with specificity 87.98%.

**Table 2:** The performance of binary-based SVM models using 10, 15 and 20 residues of N-terminus.

	**10 N-terminal residues**			**15 N-terminal residues**			**20 N-terminal residues**		
**Threshold**	**Sen.**	**Spec.**	**Acc.**	**Sen.**	**Spec.**	**Acc.**	**Sen.**	**Spec.**	**Acc.**

-1	99.75	13.55	56.65	97.95	46.55	72.25	99.10	29.85	64.48
-0.9	99.75	17.98	58.87	96.93	51.15	74.04	98.81	34.93	66.87
-0.8	99.26	25.12	62.19	96.68	57.54	77.11	98.51	44.48	71.49
-0.7	98.77	34.48	66.63	96.16	60.36	78.26	97.91	49.85	73.88
-0.6	97.04	42.86	69.95	95.65	65.22	80.43	97.01	57.91	77.46
-0.5	95.81	50.99	73.40	95.40	68.29	81.84	96.12	63.58	79.85
-0.4	94.33	59.61	76.97	93.86	73.66	83.76	94.33	69.25	81.79
-0.3	91.87	67.73	79.80	92.33	76.98	84.65	91.64	75.52	83.58
-0.2	90.64	74.38	82.51	91.05	81.33	86.19	89.55	80.00	84.78
0	**85.22**	**87.68**	**86.45**	**87.72**	**87.98**	**87.85**	**85.37**	**85.37**	**85.37**
0.1	81.28	90.39	85.84	85.93	91.30	88.62	82.69	89.85	86.27
0.2	78.33	93.60	85.96	83.63	92.84	88.24	81.79	94.03	87.91
0.3	74.63	95.07	84.85	81.59	93.86	87.72	78.81	95.82	87.31
0.4	69.21	96.80	83.00	79.03	93.86	86.45	75.82	96.72	86.27
0.5	65.76	98.03	81.90	75.70	94.63	85.17	72.24	97.31	84.78
0.6	59.85	99.01	79.43	73.40	95.40	84.40	67.46	98.21	82.84
0.7	51.48	99.51	75.49	70.08	95.65	82.86	59.70	99.10	79.40
0.8	43.10	99.75	71.43	62.92	96.42	79.67	51.34	99.10	75.22
0.9	32.27	99.75	66.01	57.29	96.93	77.11	46.57	99.40	72.99
1	25.62	99.75	62.68	52.43	97.95	75.19	37.31	99.70	68.51

#### C-terminal and N+C terminal

We have used the same strategy for the C-terminus as used for the N-terminus. The performance of binary-based SVM model using 15 C-terminal residues is shown in Table 3. Though performance was lower using C-terminal residues, the trend of performance was same. In order to check if using the N and C-termini of the peptides together will enhance the accuracy of the method, we developed a N+C-terminus based approach. In this approach the first and last 15 residues of each peptide in the original dataset were joined in order to create the positive dataset of NC-termini based method. Similar strategy as used in the N and C termini approaches were applied in this approach also. The performance of binary-based SVM model using N+C terminal residues is shown in Table 3. We got an accuracy of 92.11% with 92.11% specificity using binary-based SVM model using the first and last 15 residues. This model performs better than other models. The trend of performance in this case too follows the trend shown by the above two approaches, but the performance of this approach.

**Table 3:** The performance of binary-based SVM models using 15 N, C and N+C terminal residues.

	**N-terminal**			**C-terminal**			**N+C terminal**		
**Threshold**	**Sen.**	**Spec.**	**Acc.**	**Sen.**	**Spec.**	**Acc.**	**Sen.**	**Spec.**	**Acc.**

-1	97.95	46.55	72.25	99.74	13.80	56.77	99.07	37.59	68.33
-0.9	96.93	51.15	74.04	99.48	21.35	60.42	98.61	43.85	71.23
-0.8	96.68	57.54	77.11	99.48	28.65	64.06	98.38	51.74	75.06
-0.7	96.16	60.36	78.26	98.96	37.76	68.36	97.91	59.63	78.77
-0.6	95.65	65.22	80.43	97.66	43.75	70.70	97.45	65.89	81.67
-0.5	95.40	68.29	81.84	97.14	52.08	74.61	96.98	73.09	85.03
-0.4	93.86	73.66	83.76	95.57	59.38	77.47	96.52	78.89	87.70
-0.3	92.33	76.98	84.65	93.23	67.19	80.21	95.59	84.22	89.91
-0.2	91.05	81.33	86.19	90.36	73.44	81.90	94.43	87.47	90.95
0	**87.72**	**87.98**	**87.85**	**84.38**	**85.94**	**85.16**	**92.11**	**92.11**	**92.11**
0.1	85.93	91.30	88.62	82.03	88.28	85.16	91.18	94.66	92.92
0.2	83.63	92.84	88.24	78.91	93.49	86.20	89.33	96.06	92.69
0.3	81.59	93.86	87.72	76.04	94.79	85.42	85.85	96.29	91.07
0.4	79.03	93.86	86.45	71.61	96.61	84.11	83.99	97.45	90.72
0.5	75.70	94.63	85.17	65.36	97.66	81.51	81.67	98.14	89.91
0.6	73.40	95.40	84.40	60.68	98.96	79.82	78.19	98.61	88.40
0.7	70.08	95.65	82.86	55.21	98.96	77.08	73.78	99.54	86.66
0.8	62.92	96.42	79.67	46.09	98.96	72.53	68.21	99.54	83.87
0.9	57.29	96.93	77.11	34.38	99.74	67.06	61.02	99.77	80.39
1	52.43	97.95	75.19	24.22	100.00	62.11	51.28	99.77	75.52

#### Quantitative Matrices (QM)

In case of QM, the contribution of each residue for each position of antibacterial peptide has been calculated. A matrix with weights for each amino acid residue in every position of peptide was generated using Equation 1. Table S1 in supplementary file shows the propensity of each residue at each position in antibacterial peptides. Higher positive score of a residue at a given position means this residue is highly preferred at that position, for example G, W, D and C are highly preferred at positions 1, 2, 3, 4 and 5. Similarly, higher negative scores mean that residue is not preferred in antibacterial peptides at that position. One of the major advantages of QM is that the effect of each residue on antibacterial activity of peptide could be easily estimated. As shown in Table 4, we achieved an accuracy of 84.78%, 82.03% and 90.37%, using 15 residues of N, C and N+C terminal of peptides, respectively.

**Table 4:** The performance of QM models using 15 residues of N, C and N+C termini.

	**N-terminal**			**C-terminal**			**N+C terminal**		
**Theshold**	**Sen.**	**Spec.**	**Acc.**	**Sen.**	**Spec.**	**Acc.**	**Sen.**	**Spec.**	**Acc.**

-1	90.28	68.54	79.41	91.41	57.29	74.35	91.18	88.63	89.91
-0.9	89.77	70.84	80.31	91.15	60.42	75.78	90.72	89.33	90.02
-0.8	89.77	73.66	81.71	90.62	62.50	76.56	90.49	89.79	90.14
-0.7	89.26	75.96	82.61	90.10	66.67	78.39	90.26	90.49	90.37
-0.6	87.98	77.49	82.74	88.54	68.75	78.65	**90.02**	**90.72**	**90.37**
-0.5	87.21	79.03	83.12	87.76	71.35	79.56	89.79	92.81	91.30
-0.4	86.19	80.82	83.50	87.50	73.70	80.60	89.10	93.04	91.07
-0.3	85.42	83.12	84.27	86.46	75.26	80.86	88.40	93.74	91.07
-0.2	**84.65**	**84.91**	**84.78**	84.90	77.08	80.99	88.17	94.66	91.42
0	82.10	87.72	84.91	**81.25**	**82.81**	**82.03**	87.01	96.29	91.65
0.1	80.82	89.00	84.91	79.69	83.59	81.64	86.77	96.52	91.65
0.2	79.54	90.03	84.78	77.60	85.94	81.77	86.08	96.75	91.42
0.3	78.26	91.05	84.65	76.04	86.98	81.51	85.38	96.75	91.07
0.4	76.98	92.33	84.65	73.44	88.54	80.99	84.69	96.98	90.84
0.5	74.94	92.33	83.63	71.09	89.58	80.34	83.06	96.98	90.02
0.6	73.15	92.58	82.86	67.71	90.89	79.30	82.37	97.91	90.14
0.7	71.10	93.86	82.48	66.93	92.45	79.69	81.44	98.14	89.79
0.8	69.82	94.12	81.97	65.89	92.97	79.43	81.21	98.38	89.79
0.9	69.05	95.40	82.23	63.80	93.75	78.78	80.51	98.38	89.44
1	68.29	97.19	82.74	59.64	95.05	77.34	79.35	99.07	89.21

#### Artificial Neural Network (ANN)

The neural network is a powerful machine learning technique commonly used in the field of bioinformatics. Thus we also used this technique in this study for predicting antibacterial peptides. The performance of ANN-based method using 15 N-terminal residues is shown in Table 5. We achieved an accuracy of 83.63%, 77.34% and 88.17%, using 15 residues of N, C and N+C terminal of peptides, respectively. Though the trend was same as that of SVM, QM and ANN models, the performance of ANN was poor compared to other techniques. This is probably due to the small number of peptides used for training the models.

**Table 5:** The performance of ANN models using 15 residues of N, C and N+C termini of peptides.

	**N-terminal**			**C-terminal**			**N+C terminal**		
**Threshold**	**Sen.**	**Spec.**	**Acc.**	**Sen.**	**Spec.**	**Acc.**	**Sen.**	**Spec.**	**Acc.**

0	100.00	0.00	50.00	100.00	0.00	50.00	100.00	0.00	50.00
0.1	95.40	53.96	74.68	92.97	48.18	70.57	94.20	72.85	83.53
0.2	92.58	63.43	78.01	89.06	59.11	74.09	92.58	79.12	85.85
0.3	89.51	70.84	80.18	85.94	64.84	75.39	91.42	82.83	87.12
0.4	86.96	75.96	81.46	83.33	69.27	76.30	89.33	85.38	87.35
0.5	85.17	79.28	82.23	80.21	74.74	77.47	**88.17**	**88.17**	**88.17**
0.6	**83.38**	**83.89**	**83.63**	**76.56**	**78.12**	**77.34**	87.94	90.02	88.98
0.7	78.01	86.70	82.35	71.35	82.03	76.69	86.54	92.58	89.56
0.8	71.87	91.30	81.59	64.06	86.98	75.52	83.53	93.97	88.75
0.9	58.82	93.86	76.34	54.43	91.15	72.79	76.80	96.06	86.43
1	0.00	100.00	50.00	0.00	100.00	50.00	1.16	100.00	50.58

#### Consensus approach

In an attempt to enhance the prediction accuracy we tried the consensus approach. In this approach SVM, QM and ANN, were separately used to predict whether a peptide is antibacterial or not. A peptide was considered to be predicted positive only if at least two of these techniques predict it as a positive. Otherwise the peptide was predicted negative. The accuracy of this consensus prediction was calculated. This was done for all the three approaches i.e. N-terminal; C-terminal and NC-terminal approach (Table 6). It was observed that the performance of consensus model was slightly better than that of SVM model.

**Table 6:** Overall performance of SVM, QM and ANN based models using 15 residues of N, C and N+C-termini. Last column shows performance of and consensus approaches where two or more than two models agree.

	**SVM**			**QM**			**ANN**			**Consensus**		
**Terminus**	**Sen.**	**Spec.**	**Acc.**	**Sen.**	**Spec.**	**Acc.**	**Sen.**	**Spec.**	**Acc.**	**Sen.**	**Spec.**	**Acc.**

**N**	87.72	87.98	87.85	84.65	84.91	84.78	83.38	83.89	83.63	86.70	88.49	87.60
**C**	84.38	85.94	85.16	81.25	82.81	81.64	76.56	78.12	77.34	82.55	84.38	83.46
**N+C**	92.11	92.11	92.11	90.02	90.72	90.37	88.17	88.17	88.17	91.65	93.50	92.58

### Performance on independent or blind dataset

The models developed in this study were evaluated on an independent dataset (Table 7). These antibacterial peptides in the independent dataset were not used for developing above models either in training or testing. This dataset consists of 39 antimicrobial peptides obtained from Swiss-Prot. As shown in Table 7, SVM, ANN and QM correctly predicted 23, 19 and 21 antibacterial peptides, respectively, using N-terminal residues of peptides. In case of C-terminus based approach SVM, ANN and QM correctly predicted 30, 27 and 29 peptides, respectively. It was interesting to note that the C-terminus based models perform better than the N-terminus based models, which is in contrast to the results when evaluated using five-fold cross validation. The models based on N+C termini correctly predicted 26, 25 and 27 peptides, using SVM, ANN and QM, respectively. Overall performance of our models was very poor on independent dataset in comparison to performance on the main dataset. In order to understand the reason of poor performance we randomly extracted 39 peptides from the main dataset to form a new independent dataset and added 39 peptides from the old independent dataset to the main dataset. Then we retrained our models and evaluated their performance on 39 peptides of new independent dataset. As shown in Table 7 performance of models on these 39 peptides was as good as performance on main dataset. This shows that our 39 original peptides used as independent dataset have either some uniqueness or some problem. Thus we reanalyzed these peptides and observed that 24 out of 39 are pure peptides (only antibacterial peptides) and 15 peptides are precursors to antibacterial peptides (containing both the signal sequence and the antibacterial peptide) that require further processing in order to produce active antibacterial peptides. The poor performance on independent dataset may be due to inclusion of these precursor sequences in the independent dataset as the dataset used for training the method contains only active antibacterial peptides (and not precursors of antibacterial peptides). Thus we checked the performance of our method on these 24 active antibacterial sequences, and as expected the performance of our method on these 24 sequences was as good as on main dataset.

**Table 7:** The performance of SVM, ANN and QM models using 15 residues of N+ C termini on independent dataset.

		**SVM**		**QM**		**ANN**	
		
**Dataset**	**Terminus**	CPP*	**PPV**	**CPP**	**PPV**	**CPP**	**PPV**
**39 original peptides of independent dataset**	**N**	23	58.97	21	53.84	19	48.71
	**C**	30	76.92	29	74.35	27	69.23
	**N+C**	26	66.66	27	69.23	25	64.10
**39 randomly extracted peptides from main dataset**	**N**	35	89.74	33	84.61	34	87.17
	**C**	38	97.43	35	89.74	31	79.48
	**N+C**	36	92.30	34	87.17	36	92.30
**24 peptides obtained from original independent dataset**	**N**	21	87.50	20	83.33	17	70.83
	**C**	22	91.66	22	91.66	19	79.16
	**N+C**	22	91.66	22	91.66	22	91.66

* CPP: Correctly predicted peptides.

## Discussion

In the past attempts have been made to develop methods and strategies for designing effective antimicrobial peptides [25,26]. In this paper we have made systematic and comprehensive effort to understand and predict antibacterial peptides. As shown Figure 1 and 2, antibacterial peptides have preference for certain types of residues at certain positions. This observation is very important for experimentalists in designing de-novo antibacterial peptides; they may substitute amino acids at certain positions in order to make the peptides more effective. The quantitative matrices provide the magnitude of contribution of each type of residue at each position of antibacterial peptide. This information can be used directly for designing antibacterial peptides (See Supplementary Material). The major challenge was to develop an accurate method for predicting antibacterial peptides due to two major reasons; i) the antibacterial peptides have lot of variation in size and machine learning software need fixed length pattern and ii) experimentally proven non-antibacterial are not available which are very important for developing the method. This problem is similar to B-cell epitope prediction [27]. In order to handle the problem we developed methods using only limited number of residues from the N and C termini of peptides. In order to generate non-antibacterial peptides or negative examples, we obtained peptides randomly from non-secretory proteins [28]. Though some these peptides can be antibacterial, but chances are very rare.

First we attempted to develop models to discriminate antibacterial peptides from non-antibacterial peptides using whole peptides. In this case we used amino acid composition of peptides as input for our models. Though this model discriminates antibacterial peptides with high accuracy, it is difficult to use this model for scanning potential antibacterial proteins. Thus wee used N-terminal, C-terminal and N+C terminal approaches, as both N and C-termini play an important role in antibacterial activity, as suggested by the literature cited. The C-terminus interacts with the cell membrane and makes a pore, whereas the N-terminus helps in bacteria specific interaction process. One of the limitations of Machine Learning Techniques is that they require fixed length input vectors. Antibacterial peptides, on the contrary, are of varying length. Therefore, peptides of fixed lengths (5, 10, 15 20) were used to develop a SVM model. The number of peptides reduced significantly when 20 or 25 residue long peptides are used instead of 15 residues (335 20 mer peptides & 252 25 mer peptides). Thus in this study, we made models using 15 residues only as most (~60%) of the antibacterial peptides are 20–30 residues long. The performance of all the approaches was evaluated using five fold cross validation techniques. Performance of all modules based on QM, ANN and SVM was impressive but SVM based module using fifteen residues was the best. The poor performance of the ANN as compared to QM and SVM is due to the over optimization of ANN. It has been shown in the past that ANN suffers with the problem of over optimization when the training dataset is small [29].

AntiBP, though has some limitations like not including post-translational modifications and topological aspects, but to the best of our knowledge it is first method developed for predicting and designing potential antibacterial peptides. Our methods are likely to help the researchers in finding and in designing better peptides-based antibiotics.

## Conclusion

Currently a great deal of interest is shown in antibacterial peptides, so called "nature's antibiotics", which seem to be promising to overcome the growing problem of antibiotic resistance [30-32]. The design of novel peptides with antimicrobial activities requires the development of methods for narrowing down the candidate peptides so as to enable rational experimentation by wet-lab scientists. AntiBP is one such method meant to discover efficacious antibacterial peptides that we hope could prove to be a boon to combat the dreadful antibiotic resistant bacteria.

## Methods

### Dataset

We extracted 486 antibacterial peptides having less than 61 residues from APD database [21]. We analyzed the length of these peptides and observed that most of them have more than 15 residues. Thus we created a dataset of antibacterial peptides having number of residues between 15 and 60. After removing identical peptides, we got a dataset of 436 non-redundant antibacterial peptides, also called positive dataset. In order to generate negative dataset or dataset of non-antibacterial peptides, we extracted equal number of peptides randomly from non-secretary proteins. Our final dataset, called main dataset, has 436 antibacterial and 436 random peptides.

### N-terminal and C-terminal Dataset

In addition to the main dataset, we also created other datasets (e.g. NT5, NT10, NT15, CT15, and NTCT15) using terminal residues of peptides. In order to create a dataset NT15, we extracted 15 residues from N-terminus of antibacterial peptides. After removing identical peptides, we got 391 non-redundant peptides of length 15. An equal number of random peptides have been extracted from non-secretory proteins. Finally, dataset NT15 has 391 antibacterial peptides and 391 random peptides of length 15. Similarly, we created datasets NT5 and NT10 after extracting 5 and 10 residues from N-terminus of antibacterial peptides. In order to create dataset CT15, we extracted 15 residues from the C-terminus of antibacterial peptides. After removing identical peptides, we got 384 non-redundant antibacterial peptides and an equal number of random peptides. Similarly, we created datasets CT5 and CT10. In order to create dataset NTCT15, we extracted 15 residues from N-terminus and 15-residues from C-terminus of antibacterial peptides. After removing identical peptides, we got 431 non-redundant antibacterial peptides; equal number of random peptides was extracted from non-secretory proteins.

### Blind or Independent dataset

All antibacterial peptides with residues between 15 and 80 were extracted from Swiss-Prot database and from this set peptides present in our main dataset were removed. Finally we got a dataset of 39 antibacterial peptides called blind and independent dataset. All these 39 peptides are experimentally verified and annotated as antibacterial peptides according to Swiss-Prot.

### Quantitative matrix

The quantitative matrix is basically a propensity of each residue at a particular position. To classify the data of antibacterial peptides and non-antibacterial peptides, different quantitative matrices were generated for the N-terminal and C-terminal residues. Following equation was used to generate the quantitative matrices

Q _(i,r) _= P _(i,r) _- N _(i,r)_

P(i,r)=Ei,rNPi,r
 MathType@MTEF@5@5@+=feaafiart1ev1aaatCvAUfKttLearuWrP9MDH5MBPbIqV92AaeXatLxBI9gBaebbnrfifHhDYfgasaacH8akY=wiFfYdH8Gipec8Eeeu0xXdbba9frFj0=OqFfea0dXdd9vqai=hGuQ8kuc9pgc9s8qqaq=dirpe0xb9q8qiLsFr0=vr0=vr0dc8meaabaqaciaacaGaaeqabaqabeGadaaakeaacqqGqbaudaWgaaWcbaGaeiikaGIaeeyAaKMaeeilaWIaeeOCaiNaeiykaKcabeaakiabg2da9eaadaWcaaqaaiabdweafnaaBaaaleaacqWGPbqAcqWGSaalcqWGYbGCaeqaaaGcbaGaemOta4Kaemiuaa1aaSbaaSqaaiabdMgaPjabdYcaSiabdkhaYbqabaaaaaaaaa@3F8A@

N(i,r)=Ai,rNNi,r
 MathType@MTEF@5@5@+=feaafiart1ev1aaatCvAUfKttLearuWrP9MDH5MBPbIqV92AaeXatLxBI9gBaebbnrfifHhDYfgasaacH8akY=wiFfYdH8Gipec8Eeeu0xXdbba9frFj0=OqFfea0dXdd9vqai=hGuQ8kuc9pgc9s8qqaq=dirpe0xb9q8qiLsFr0=vr0=vr0dc8meaabaqaciaacaGaaeqabaqabeGadaaakeaacqqGobGtdaWgaaWcbaGaeiikaGIaeeyAaKMaeeilaWIaeeOCaiNaeiykaKcabeaakiabg2da9eaadaWcaaqaaiabdgeabnaaBaaaleaacqWGPbqAcqWGSaalcqWGYbGCaeqaaaGcbaGaemOta4KaemOta40aaSbaaSqaaiabdMgaPjabdYcaSiabdkhaYbqabaaaaaaaaa@3F7A@

Where, Q_(i,r) _is the weight of residue r at position 'i' in the matrix. 'r' can be any natural amino acid and the value of 'i' can vary from 1 to 15. P_(i,r) _and N_(i,r) _is the probability of residue 'r' at position 'i' in antibacterial peptides and non-antibacterial peptides respectively. E_i,r _and A_i,r _is number residue 'r' at position 'i' in antibacterial peptides and non-antibacterial peptides, respectively, and NP_i,r _is the number of antibacterial peptides and NN_i,r _is the number of non-antibacterial peptides having residue 'r' at position 'i'. The quantitative matrix generated by using eq.(1), which is an addition matrix where the score of a peptide is calculated by summing up the scores of each residue at specific position along peptide sequence as

Score=∑i=1LQ(i,r)
 MathType@MTEF@5@5@+=feaafiart1ev1aaatCvAUfKttLearuWrP9MDH5MBPbIqV92AaeXatLxBI9gBaebbnrfifHhDYfgasaacH8akY=wiFfYdH8Gipec8Eeeu0xXdbba9frFj0=OqFfea0dXdd9vqai=hGuQ8kuc9pgc9s8qqaq=dirpe0xb9q8qiLsFr0=vr0=vr0dc8meaabaqaciaacaGaaeqabaqabeGadaaakeaacqqGtbWucqqGJbWycqqGVbWBcqqGYbGCcqqGLbqzcqGH9aqpdaaeWbqaaiabbgfarnaaBaaaleaacqGGOaakcqqGPbqAcqGGSaalcqqGYbGCcqGGPaqkaeqaaaqaaiabbMgaPjabg2da9iabigdaXaqaaiabbYeambqdcqGHris5aaaa@419D@

Where, L is the length of the peptide.

### Support Vector Machine

In this study, all SVM models have been developed using a freely available program SVM_LIGHT [33]. This program allows users to run SVM using various kernels and parameters. In this study, the accuracy was computed at a cut-off score where sensitivity and specificity are nearly equal.

### Artificial Neural network

In order to develop ANN models we used Stuttgart Neural Network Simulator, SNNS version 4.2 [34]. The advantage of this package is that it allows incorporation of resulting networks in ANSI C functions for use in stand-alone code. The critical step in ANN is the optimization of the hidden nodes and other learning parameters, in order to achieve the best performance. Standard square error (SSE) was used for the performance function. The number of epochs has been taken where the value of SSE was minimum. For N-terminal method we used the feed forward back propagation type of ANN with single hidden layer having 17 nodes, 300 (20 × 15) input units and 1 output unit. The training was carried out for 20000 epochs. Where as for C-terminal approach we have used the feed forward back propagation type of ANN with single hidden layer having 25 nodes, 300 (20 × 15) input units and 1 output unit. Training was carried out for 10000 epochs. For N+C-terminal approach feed forward back propagation ANN with single layer having 25 nodes, 600 (20 × 30) input units and a 1 output unit were used.

### Evaluation of the Method

Five-fold cross-validation technique has been used to evaluate the performance of all the models developed in this study. In five fold cross-validation technique a dataset is randomly divided into five sets, where each set consists of nearly equal number of antibacterial peptides and non antibacterial peptides. Four sets are used for training and the remaining set for testing. This process is repeated five times so that each set is used once for testing. The performance of method is average performance of method on five sets. Following parameters has been used for assessing the performance of a method.

Sensitivity=TPTP+FN×100
 MathType@MTEF@5@5@+=feaafiart1ev1aaatCvAUfKttLearuWrP9MDH5MBPbIqV92AaeXatLxBI9gBaebbnrfifHhDYfgasaacH8akY=wiFfYdH8Gipec8Eeeu0xXdbba9frFj0=OqFfea0dXdd9vqai=hGuQ8kuc9pgc9s8qqaq=dirpe0xb9q8qiLsFr0=vr0=vr0dc8meaabaqaciaacaGaaeqabaqabeGadaaakeaacqqGtbWucqqGLbqzcqqGUbGBcqqGZbWCcqqGPbqAcqqG0baDcqqGPbqAcqqG2bGDcqqGPbqAcqqG0baDcqqG5bqEiiaacqWF9aqpbaWaaSaaaeaacqWGubavcqWGqbauaeaacqWGubavcqWGqbaucqWFRaWkcqWGgbGrcqWGobGtaaGaey41aqRaeeymaeJaeeimaaJaeeimaadaaaa@4980@

Specificity=TNTN+FP×100
 MathType@MTEF@5@5@+=feaafiart1ev1aaatCvAUfKttLearuWrP9MDH5MBPbIqV92AaeXatLxBI9gBaebbnrfifHhDYfgasaacH8akY=wiFfYdH8Gipec8Eeeu0xXdbba9frFj0=OqFfea0dXdd9vqai=hGuQ8kuc9pgc9s8qqaq=dirpe0xb9q8qiLsFr0=vr0=vr0dc8meaabaqaciaacaGaaeqabaqabeGadaaakeaacqqGtbWucqqGWbaCcqqGLbqzcqqGJbWycqqGPbqAcqqGMbGzcqqGPbqAcqqGJbWycqqGPbqAcqqG0baDcqqG5bqEiiaacqWF9aqpbaWaaSaaaeaacqWGubavcqWGobGtaeaacqWGubavcqWGobGtcqWFRaWkcqWGgbGrcqWGqbauaaGaey41aqRaeeymaeJaeeimaaJaeeimaadaaaa@491E@

Accuracy=TP+TNTP+FP+TN+FN×100
 MathType@MTEF@5@5@+=feaafiart1ev1aaatCvAUfKttLearuWrP9MDH5MBPbIqV92AaeXatLxBI9gBaebbnrfifHhDYfgasaacH8akY=wiFfYdH8Gipec8Eeeu0xXdbba9frFj0=OqFfea0dXdd9vqai=hGuQ8kuc9pgc9s8qqaq=dirpe0xb9q8qiLsFr0=vr0=vr0dc8meaabaqaciaacaGaaeqabaqabeGadaaakeaabaGaeeyqaeKaee4yamMaee4yamMaeeyDauNaeeOCaiNaeeyyaeMaee4yamMaeeyEaKhccaGae8xpa0ZaaSaaaeaacqWGubavcqWGqbaucqWFRaWkcqWGubavcqWGobGtaeaacqWGubavcqWGqbaucqWFRaWkcqWGgbGrcqWGqbaucqWFRaWkcqWGubavcqWGobGtcqWFRaWkcqWGgbGrcqWGobGtaaGaey41aqRaeGymaeJaeGimaaJaeGimaadaaaa@4E7B@

PPV=TPTP+FP×100
 MathType@MTEF@5@5@+=feaafiart1ev1aaatCvAUfKttLearuWrP9MDH5MBPbIqV92AaeXatLxBI9gBaebbnrfifHhDYfgasaacH8akY=wiFfYdH8Gipec8Eeeu0xXdbba9frFj0=OqFfea0dXdd9vqai=hGuQ8kuc9pgc9s8qqaq=dirpe0xb9q8qiLsFr0=vr0=vr0dc8meaabaqaciaacaGaaeqabaqabeGadaaakeaacqqGqbaucqqGqbaucqqGwbGvcqGH9aqpdaWcaaqaaiabdsfaujabdcfaqbqaaiabdsfaujabdcfaqHGaaiab=TcaRiabdAeagjabdcfaqbaacqGHxdaTcqaIXaqmcqaIWaamcqaIWaamaaa@3DFD@, where PPV is Probability of positive prediction.

Where TP and TN are correctly predicted antibacterial peptides and non-antibacterial peptides respectively. FP and FN are wrongly predicted antibacterial peptides and non – antibacterial peptides respectively. The five fold cross validation technique was used for evaluation of all the three methods used.

## Availability and requirements

We developed a web server AntiBP available from  for predicting bacterial peptides using models developed in this study. This web server was developed on SUN server (model T-1000) under Solaris environment using programming language PERL. It also allows mapping and searching of antibacterial in a protein sequence. This server is free for academic use, commercials should contact author for licence.

## Authors' contributions

SL and BK created dataset and developed the web site. SL developed SVM and QM models and BK developed ANN models. GPSR conceived the project, coordinated it and refined the manuscript drafted by SL.

## Supplementary Material

Additional file 1 Supplementary file. supplementary file carries the detailed analysis of the antibacterial peptides and tabulates performance of the models when different lengths of peptides from N and C termini were used. Click here for file
