# Supplementary material for: Analysis and prediction of antibacterial peptides
Source: BMC Bioinformatics. 2007 Jul 23;8:263. doi: 10.1186/1471-2105-8-263 (PMC2041956; doi:10.1186/1471-2105-8-263)
Supplement: Additional file 1 — Supplementary file. supplementary file carries the detailed analysis of the antibacterial peptides and tabulates performance of the models when different lengths of peptides from N and C termini were used. [file 1471-2105-8-263-S1.doc]

**Supplementary Material**

**Title:** Analysis and Prediction of Anti-bacterial Peptides

# Authors: Sneh Lata, B. K. Sharma and G. P. S. Raghava*; Institute of Microbial Technology, Sector-39A, Chandigarh, India

**Figure S1:** Analysis of residues at first position of N-terminus of antibacterial peptides. Height of bar shows the frequency of residues at a position.

**Figure S2:** Analysis of residues at 2nd position of N-terminus of antibacterial peptides. Height of bar shows the frequency of residues at a position.

**Figure S3:** Analysis of residues at 3rd position of N-terminus of antibacterial peptides. Height of bar shows the frequency of residues at a position.

**Figure S4:** Analysis of residues at 4th position of N-terminus of antibacterial peptides. Height of bar shows the frequency of residues at a position.

**Figure S5:** Analysis of residues at 5th position of N-terminus of antibacterial peptides. Height of bar shows the frequency of residues at a position.

**Figure S6:** Analysis of residues at 1^st^ position of C-terminus of antibacterial peptides. Height of bar shows the frequency of residues at a position. **Figure S7:** Analysis of residues at 2nd position of C-terminus of antibacterial peptides. Height of bar shows the frequency of residues at a position.

**Figure S8:** Analysis of residues at 3rd position of C-terminus of antibacterial peptides. Height of bar shows the frequency of residues at a position.

**Figure S9:** Analysis of residues at 4th position of C-terminus of antibacterial peptides. Height of bar shows the frequency of residues at a position.

**Figure S10:** Analysis of residues at 5th position of C-terminus of antibacterial peptides. Height of bar shows the frequency of residues at a position.

**Figure S11:** Frequency of polar, non-polar, negative charge and C+R+K in antibacterial and non-antibacterial peptides at 1^st^ position of N-terminus.

**Figure S12:** Frequency of polar, non-polar, negative charge and C+R+K in antibacterial and non-antibacterial peptides at 2nd position of N-terminus.

**Figure S13:** Frequency of polar, non-polar, negative charge and C+R+K in antibacterial and non-antibacterial peptides at 2nd position of N-terminus.

**Figure S14:** Frequency of polar, non-polar, negative charge and C+R+K in antibacterial and non-antibacterial peptides at 4th position of N-terminus.

**Figure S15:** Frequency of polar, non-polar, negative charge and C+R+K in antibacterial and non-antibacterial peptides at 5th position of N-terminus.

**Figure S16:** Frequency of polar, non-polar, negative charge and C+R+K in antibacterial and non-antibacterial peptides at 1st position of C-terminus.

**Figure S17:** Frequency of polar, non-polar, negative charge and C+R+K in antibacterial and non-antibacterial peptides at 2nd position of C-terminus.

**Figure S18:** Frequency of polar, non-polar, negative charge and C+R+K in antibacterial and non-antibacterial peptides at 3rd position of C-terminus.

**Figure S19:** Frequency of polar, non-polar, negative charge and C+R+K in antibacterial and non-antibacterial peptides at 4^th^ position of C-terminus.

**Figure S20:** Frequency of polar, non-polar, negative charge and C+R+K in antibacterial and non-antibacterial peptides at 5th position of C-terminus.

**Figure S21:** Number of antibacterial peptides of various lengths in original dataset.

**Figure S22**: creation of NT15, CT15 and NTCT15 datasets.


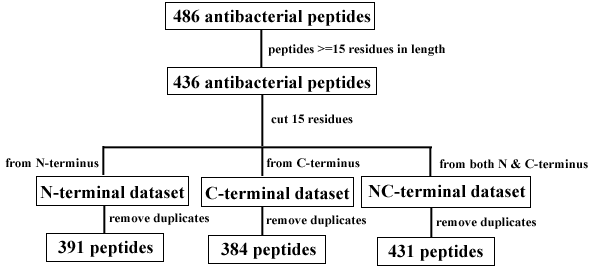


**Figure S23**: Creation of NT5 dataset.


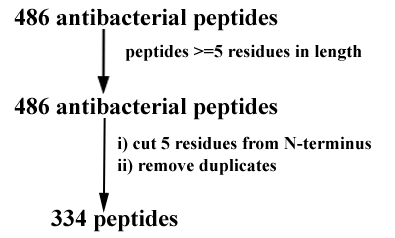


**Figure S24**: Creation of NT10 dataset.


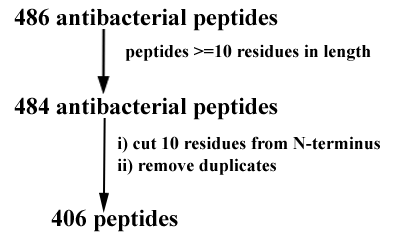


**Figure S25:** Creation of NT20 dataset.


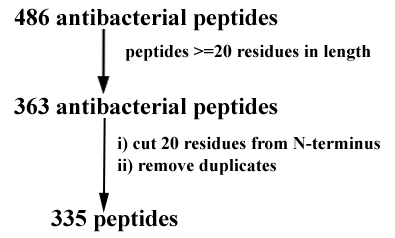


**Table S1:** Quantitative weight matrix for first fifteen residues of N-terminus of antibacterial peptides. P1, P2... P15 shows residue preferences for positions 1, 2... 15, respectively. The number shown in bold has highest propensity of a residue in a given position.

| AA | P1 | P2 | P3 | P4 | P5 | P6 | P7 | P8 | P9 | P10 | P11 | P12 | P13 | P14 | P15 |
| --- | --- | --- | --- | --- | --- | --- | --- | --- | --- | --- | --- | --- | --- | --- | --- |
| A | -0.241 | -0.600 | -0.641 | -0.030 | 0.135 | -0.014 | -0.261 | -0.091 | 0.168 | 0.477 | -0.074 | -0.156 | -0.028 | 0.333 | 0.276 |
| C | -0.231 | 0.765 | 0.467 | 0.379 | **0.556** | 0.333 | 0.185 | 0.444 | **0.586** | 0.429 | 0.083 | 0.333 | 0.333 | 0.133 | 0.167 |
| D | -0.120 | -0.294 | -0.400 | **0.531** | -0.059 | *-0.750* | -0.765 | -0.250 | -0.500 | *-0.875* | -0.529 | -0.524 | -0.786 | -0.556 | *-0.905* |
| E | *-0.882* | *-0.889* | -0.556 | -0.429 | -0.829 | -0.500 | -0.812 | *-0.562* | -0.368 | -0.800 | -0.317 | -0.394 | -0.471 | -0.282 | -0.742 |
| F | 0.472 | 0.488 | 0.283 | 0.467 | 0.442 | 0.027 | -0.455 | -0.267 | 0.097 | -0.154 | -0.462 | 0.231 | 0.333 | -0.500 | 0.056 |
| G | **0.695** | -0.111 | 0.470 | 0.082 | -0.318 | -0.048 | 0.432 | 0.100 | 0.129 | **0.486** | 0.434 | 0.229 | 0.065 | 0.310 | 0.036 |
| H | -0.200 | -0.538 | -0.263 | -0.538 | *-0.833* | -0.412 | -0.111 | -0.176 | 0.111 | 0.385 | **0.667** | 0.500 | **0.417** | -0.176 | 0.000 |
| I | 0.000 | 0.590 | 0.111 | 0.062 | 0.281 | 0.532 | -0.185 | 0.347 | 0.191 | 0.000 | 0.125 | -0.185 | 0.288 | -0.200 | -0.040 |
| K | -0.240 | -0.304 | 0.236 | 0.137 | 0.270 | 0.097 | **0.678** | **0.471** | 0.515 | 0.164 | 0.613 | **0.576** | 0.167 | **0.377** | **0.569** |
| L | -0.485 | 0.411 | -0.055 | -0.171 | 0.059 | 0.333 | 0.039 | -0.318 | -0.229 | -0.333 | -0.472 | -0.333 | 0.191 | -0.023 | -0.404 |
| M | -0.636 | -0.429 | -0.333 | -0.250 | -0.500 | **0.600** | *-1.000* | 0.091 | -0.200 | -0.750 | -0.800 | -0.333 | 0.077 | *-1.000* | -0.455 |
| N | -0.667 | -0.048 | -0.176 | -0.300 | 0.000 | -0.619 | -0.429 | 0.238 | -0.600 | -0.217 | 0.026 | 0.067 | -0.300 | 0.067 | -0.105 |
| P | -0.852 | -0.643 | 0.216 | 0.050 | -0.314 | -0.381 | -0.438 | -0.273 | -0.314 | 0.250 | -0.467 | -0.098 | -0.394 | 0.294 | -0.037 |
| Q | -0.278 | -0.806 | -0.524 | -0.533 | -0.556 | -0.500 | -0.517 | -0.333 | -0.583 | -0.586 | -0.600 | -0.636 | -0.355 | -0.500 | -0.238 |
| R | 0.244 | -0.064 | -0.091 | 0.127 | 0.055 | -0.023 | 0.414 | 0.036 | 0.133 | 0.048 | 0.240 | 0.000 | 0.244 | 0.200 | 0.208 |
| S | -0.365 | -0.423 | -0.368 | 0.275 | -0.207 | -0.548 | -0.038 | -0.068 | -0.422 | -0.500 | -0.059 | -0.164 | -0.444 | -0.636 | 0.118 |
| T | -0.600 | -0.111 | *-0.667* | -0.111 | 0.048 | -0.167 | -0.353 | -0.027 | *-0.652* | -0.241 | -0.333 | 0.053 | -0.562 | -0.440 | -0.077 |
| V | 0.034 | -0.300 | -0.167 | *-0.667* | 0.088 | 0.000 | -0.442 | -0.250 | 0.273 | 0.148 | -0.190 | 0.188 | 0.211 | -0.191 | -0.086 |
| W | -0.111 | **0.778** | **0.500** | -0.273 | 0.091 | -0.111 | -0.500 | -0.143 | -0.250 | -0.556 | 0.200 | 0.000 | *-1.000* | -0.400 | -0.667 |
| Y | -0.273 | -0.429 | -0.143 | -0.636 | -0.333 | 0.000 | 0.364 | -0.222 | -0.500 | -0.176 | *-1.000* | *-0.765* | 0.125 | -0.053 | -0.529 |

**Table S2:** Quantitative weight matrix for last fifteen residues of C-terminus of antibacterial peptides. P1, P2. P15 shows residue preferences for positions 1, 2 … 15 respectively. The number shown in bold has highest propensity of a residue in a given position.

| AA | P1 | P2 | P3 | P4 | P5 | P6 | P7 | P8 | P9 | P10 | P11 | P12 | P13 | P14 | P15 |
| --- | --- | --- | --- | --- | --- | --- | --- | --- | --- | --- | --- | --- | --- | --- | --- |
| A | -0.320 | -0.016 | -0.051 | 0.205 | 0.375 | 0.108 | -0.160 | 0.167 | 0.012 | 0.217 | 0.134 | -0.072 | -0.059 | 0.413 | 0.455 |
| C | **0.775** | **0.871** | 0.520 | **0.692** | **0.636** | **0.471** | **0.676** | 0.167 | 0.294 | 0.238 | 0.389 | **0.517** | 0.273 | 0.161 | 0.167 |
| D | *-0.867* | -0.375 | *-0.857* | -0.579 | *-0.565* | -0.625 | -0.500 | *-0.579* | *-0.833* | -0.200 | -0.412 | -0.333 | -0.190 | -0.167 | -0.538 |
| E | -0.185 | -0.172 | -0.167 | *-0.622* | -0.409 | *-0.739* | -0.758 | -0.562 | -0.724 | -0.636 | -0.576 | *-0.630* | *-0.714* | *-0.714* | *-0.857* |
| F | 0.097 | *-0.467* | -0.226 | 0.059 | -0.143 | -0.565 | -0.231 | 0.116 | 0.125 | -0.200 | 0.000 | -0.081 | 0.200 | 0.217 | -0.214 |
| G | 0.037 | 0.049 | 0.228 | -0.244 | -0.487 | 0.362 | 0.148 | **0.257** | 0.224 | 0.367 | 0.194 | 0.239 | 0.222 | 0.349 | 0.224 |
| H | 0.000 | 0.200 | -0.043 | 0.091 | -0.429 | 0.143 | 0.200 | -0.385 | 0.273 | **0.467** | **0.625** | 0.000 | 0.125 | 0.048 | -0.143 |
| I | -0.032 | -0.103 | -0.143 | 0.231 | 0.410 | 0.273 | 0.362 | 0.256 | 0.283 | -0.048 | -0.050 | 0.360 | 0.222 | 0.100 | 0.091 |
| K | 0.016 | 0.310 | **0.548** | 0.403 | 0.098 | 0.317 | 0.356 | 0.148 | **0.500** | 0.289 | 0.345 | 0.391 | **0.524** | **0.415** | 0.462 |
| L | -0.067 | -0.464 | -0.487 | -0.108 | 0.000 | -0.207 | 0.089 | -0.018 | -0.051 | -0.395 | -0.068 | 0.178 | 0.013 | -0.075 | -0.026 |
| M | -0.500 | -0.250 | -0.273 | -0.429 | 0.000 | 0.000 | -0.273 | -0.250 | -0.333 | -0.167 | -0.800 | -0.231 | -0.333 | -0.333 | -0.600 |
| N | 0.600 | 0.214 | 0.200 | 0.000 | 0.167 | -0.360 | -0.200 | 0.059 | -0.091 | 0.212 | -0.333 | -0.474 | -0.625 | -0.077 | -0.615 |
| P | -0.351 | -0.179 | -0.212 | -0.152 | -0.222 | -0.137 | -0.128 | -0.366 | -0.070 | 0.091 | 0.222 | 0.057 | -0.353 | -0.455 | -0.366 |
| Q | 0.061 | -0.256 | -0.032 | -0.353 | -0.167 | -0.263 | -0.571 | -0.333 | -0.188 | -0.438 | -0.231 | -0.286 | -0.556 | -0.125 | 0.037 |
| R | 0.443 | 0.352 | 0.238 | -0.022 | -0.300 | -0.077 | 0.111 | 0.129 | 0.000 | 0.073 | 0.026 | -0.333 | 0.189 | -0.375 | 0.208 |
| S | -0.433 | -0.298 | -0.412 | 0.000 | -0.346 | -0.333 | -0.421 | -0.357 | -0.455 | -0.255 | -0.061 | -0.550 | -0.351 | -0.149 | -0.098 |
| T | -0.412 | 0.097 | -0.081 | 0.268 | -0.290 | -0.143 | -0.353 | -0.357 | -0.226 | 0.053 | -0.143 | -0.133 | -0.163 | -0.286 | -0.037 |
| V | -0.400 | -0.182 | -0.227 | -0.360 | 0.225 | 0.028 | 0.048 | 0.250 | 0.176 | 0.164 | 0.000 | 0.067 | 0.156 | -0.400 | -0.226 |
| W | 0.286 | -0.333 | -0.200 | -0.750 | 0.091 | -0.429 | *-1.000* | 0.111 | -0.667 | *-1.000* | *-0.600* | -0.333 | -0.600 | -0.556 | -0.667 |
| Y | 0.125 | 0.048 | -0.043 | -0.385 | 0.167 | 0.100 | 0.263 | 0.043 | -0.412 | 0.167 | -0.455 | 0.032 | -0.077 | -0.250 | -0.238 |

**Table S3:** Performance of SVM module developed by using amino acid composition and binary pattern of NT5 dataset.

|  | **Amino acid composition** | | | **Binary pattern** | | |
| --- | --- | --- | --- | --- | --- | --- |
|  |  |  |  |  |  |  |
| **Theshold** | **Sen. (%)** | **Spec. (%)** | **Acc. (%)** | **Sen. (%)** | **Spec. (%)** | **Acc. (%)** |
| -1 | 97.31 | 25.45 | 61.38 | 95.81 | 24.25 | 60.03 |
| -0.9 | 95.21 | 30.54 | 62.87 | 94.31 | 30.54 | 62.43 |
| -0.8 | 93.71 | 35.33 | 64.52 | 93.71 | 37.13 | 65.42 |
| -0.7 | 91.62 | 39.82 | 65.72 | 91.62 | 43.11 | 67.37 |
| -0.6 | 90.42 | 46.11 | 68.26 | 90.12 | 48.50 | 69.31 |
| -0.5 | 89.22 | 50.60 | 69.91 | 86.83 | 54.19 | 70.51 |
| -0.4 | 87.72 | 55.09 | 71.41 | 85.03 | 59.88 | 72.46 |
| -0.3 | 84.73 | 58.98 | 71.86 | 82.34 | 64.97 | 73.65 |
| -0.2 | 81.44 | 63.47 | 72.46 | 80.24 | 68.86 | 74.55 |
| 0 | **73.65** | **72.75** | **73.20** | **74.25** | **74.85** | **74.55** |
| 0.1 | 70.36 | 76.65 | 73.50 | 70.66 | 77.54 | 74.10 |
| 0.2 | 66.77 | 79.64 | 73.20 | 67.96 | 83.83 | 75.90 |
| 0.3 | 63.47 | 83.83 | 73.65 | 64.97 | 88.32 | 76.65 |
| 0.4 | 57.49 | 86.83 | 72.16 | 60.78 | 88.92 | 74.85 |
| 0.5 | 53.59 | 89.52 | 71.56 | 57.78 | 90.12 | 73.95 |
| 0.6 | 48.50 | 91.62 | 70.06 | 54.49 | 91.02 | 72.75 |
| 0.7 | 44.61 | 94.01 | 69.31 | 51.50 | 93.41 | 72.46 |
| 0.8 | 39.22 | 94.91 | 67.07 | 44.91 | 95.51 | 70.21 |
| 0.9 | 34.43 | 96.11 | 65.27 | 39.52 | 96.11 | 67.81 |
| 1 | 29.04 | 97.90 | 63.47 | 32.34 | 97.01 | 64.67 |

**Table S4:** Performance of SVM module developed by using amino acid composition and binary pattern of NT10 dataset.

|  | **Amino acid composition** | | | **Binary pattern** | | |
| --- | --- | --- | --- | --- | --- | --- |
|  |  |  |  |  |  |  |
| **Theshold** | **Sen. (%)** | **Spec. (%)** | **Acc. (%)** | **Sen. (%)** | **Spec. (%)** | **Acc. (%)** |
| -1 | 97.29 | 44.33 | 70.81 | 99.75 | 13.55 | 56.65 |
| -0.9 | 97.04 | 49.01 | 73.03 | 99.75 | 17.98 | 58.87 |
| -0.8 | 95.81 | 52.46 | 74.14 | 99.26 | 25.12 | 62.19 |
| -0.7 | 94.58 | 55.91 | 75.25 | 98.77 | 34.48 | 66.63 |
| -0.6 | 93.60 | 59.61 | 76.60 | 97.04 | 42.86 | 69.95 |
| -0.5 | 92.86 | 63.05 | 77.96 | 95.81 | 50.99 | 73.40 |
| -0.4 | 91.87 | 65.52 | 78.69 | 94.33 | 59.61 | 76.97 |
| -0.3 | 90.89 | 67.73 | 79.31 | 91.87 | 67.73 | 79.80 |
| -0.2 | 89.66 | 72.17 | 80.91 | 90.64 | 74.38 | 82.51 |
| 0 | **83.74** | **81.28** | **82.51** | **85.22** | **87.68** | **86.45** |
| 0.1 | 81.03 | 83.25 | 82.14 | 81.28 | 90.39 | 85.84 |
| 0.2 | 78.82 | 85.22 | 82.02 | 78.33 | 93.60 | 85.96 |
| 0.3 | 75.12 | 87.68 | 81.40 | 74.63 | 95.07 | 84.85 |
| 0.4 | 70.94 | 90.89 | 80.91 | 69.21 | 96.80 | 83.00 |
| 0.5 | 68.97 | 91.38 | 80.17 | 65.76 | 98.03 | 81.90 |
| 0.6 | 64.29 | 93.10 | 78.69 | 59.85 | 99.01 | 79.43 |
| 0.7 | 58.37 | 93.84 | 76.11 | 51.48 | 99.51 | 75.49 |
| 0.8 | 53.94 | 94.58 | 74.26 | 43.10 | 99.75 | 71.43 |
| 0.9 | 49.51 | 96.31 | 72.91 | 32.27 | 99.75 | 66.01 |
| 1 | 45.57 | 96.55 | 71.06 | 25.62 | 99.75 | 62.68 |

**Table S5:** Performance of SVM module developed by using amino acid composition and binary pattern of NT15 dataset.

|  | **Amino acid composition** | | | **Binary pattern** | | |
| --- | --- | --- | --- | --- | --- | --- |
|  |  |  |  |  |  |  |
| **Theshold** | **Sen. (%)** | **Spec. (%)** | **Acc. (%)** | **Sen. (%)** | **Spec. (%)** | **Acc. (%)** |
| -1 | 96.93 | 51.66 | 74.30 | 97.95 | 46.55 | 72.25 |
| -0.9 | 96.68 | 56.52 | 76.60 | 96.93 | 51.15 | 74.04 |
| -0.8 | 96.42 | 62.40 | 79.41 | 96.68 | 57.54 | 77.11 |
| -0.7 | 95.91 | 65.98 | 80.95 | 96.16 | 60.36 | 78.26 |
| -0.6 | 94.63 | 68.29 | 81.46 | 95.65 | 65.22 | 80.43 |
| -0.5 | 93.61 | 71.61 | 82.61 | 95.40 | 68.29 | 81.84 |
| -0.4 | 92.07 | 76.21 | 84.14 | 93.86 | 73.66 | 83.76 |
| -0.3 | 91.05 | 78.77 | 84.91 | 92.33 | 76.98 | 84.65 |
| -0.2 | 90.03 | 81.33 | 85.68 | 91.05 | 81.33 | 86.19 |
| 0 | **88.24** | **88.24** | **88.24** | **87.72** | **87.98** | **87.85** |
| 0.1 | 86.45 | 89.77 | 88.11 | 85.93 | 91.30 | 88.62 |
| 0.2 | 84.40 | 90.79 | 87.60 | 83.63 | 92.84 | 88.24 |
| 0.3 | 81.33 | 92.58 | 86.96 | 81.59 | 93.86 | 87.72 |
| 0.4 | 78.52 | 94.12 | 86.32 | 79.03 | 93.86 | 86.45 |
| 0.5 | 77.49 | 94.37 | 85.93 | 75.70 | 94.63 | 85.17 |
| 0.6 | 73.66 | 95.65 | 84.65 | 73.40 | 95.40 | 84.40 |
| 0.7 | 70.59 | 96.16 | 83.38 | 70.08 | 95.65 | 82.86 |
| 0.8 | 67.26 | 96.68 | 81.97 | 62.92 | 96.42 | 79.67 |
| 0.9 | 62.92 | 97.70 | 80.31 | 57.29 | 96.93 | 77.11 |
| 1 | 57.80 | 97.70 | 77.75 | 52.43 | 97.95 | 75.19 |
